# Supplementary material for: Promoting or pressurising participation? A discourse analysis of online patient information resources about prehabilitation before cancer treatment
Source: PLoS One. 2025 Dec 23;20(12):e0339169. doi: 10.1371/journal.pone.0339169 (PMC12725656; doi:10.1371/journal.pone.0339169)
Supplement: S1 Appendix — (DOCX) [file pone.0339169.s001.docx]

**Database: TRIP Pro**

| **#** | **Search Terms** | **Results** | **Screened** | **Potentially relevant records** |
| --- | --- | --- | --- | --- |
| 0a | (("pre habilitation") OR ("pre-habilitation") OR (prehab*) OR (prehabilitation)) | 1533 🡪 2 with patient information only filter | 2 | 0 |
| 0b | ((patient information) OR ("information for patients") OR ("patient information")) AND ((("pre habilitation") OR ("pre-habilitation") OR (prehab*) OR (prehabilitation))) | 622 🡪 2 with patient information only filter | 2 | 0 |
| 0c | (cancer) AND ((("pre habilitation") OR ("pre-habilitation") OR (prehab*) OR (prehabilitation))) | 752 🡪 1  with patient information only filter | 1 | 0 |
| 0d | (cancer) AND (((patient information) OR ("information for patients") OR ("patient information")) AND ((("pre habilitation") OR ("pre-habilitation") OR (prehab*) OR (prehabilitation)))) | 368 🡪 1 with patient information only filter | 1 | 0 |

**Search Engine: Google**

| **#** | **Domain** | **Search Terms** | **Results** | **Screened** | **Potentially relevant records** |
| --- | --- | --- | --- | --- | --- |
| 1a | *.nhs.uk  *.nhs.wales  *.hscni.net | Prehabilitation OR prehab* OR "pre-habilitation" OR "pre habilitation" | 12,400 | 100 | 46 |
| 1b | *.org.uk |  | 4350 | 100 | 4 |
| 1c | *.ac.uk |  | 8790 | 100 | 1 |
| 1d | No restriction (UK only) |  | 64,300 | 100 | 9 |
| 2a | *.nhs.uk  *.nhs.wales  *.hscni.net | Prehabilitation OR prehab* OR "pre-habilitation" OR "pre habilitation" AND "patient information" OR “information for patients” | 6,180 | 100 | 40 |
| 2b | *.org.uk |  | 925 | 100 | 4 |
| 2c | *.ac.uk |  | 2,390 | 100 | 0 |
| 2d | No restriction |  | 13,900 | 100 | 19 |
| 3a | *.nhs.uk  *.nhs.wales  *.hscni.net | Prehabilitation OR prehab* OR "pre-habilitation" OR "pre habilitation" AND cancer | 11,300 | 100 | 6 |
| 3b | *.org.uk |  | 3,920 | 100 | 0 |
| 3c | *.ac.uk |  | 4,500 | 100 | 0 |
| 3d | No restriction |  | 58,800 | 100 | 2 |
| 4a | *.nhs.uk  *.nhs.wales  *.hscni.net | Prehabilitation OR prehab* OR "pre-habilitation" OR "pre habilitation" AND "patient information" OR “information for patients” AND cancer | 5,880 | 100 | 4 |
| 4b | *.org.uk |  | 701 | 100 | 4 |
| 4c | *.ac.uk |  | 750 | 100 | 0 |
| 4d | No restriction |  | 10,200 | 100 | 4 |

**Search Engine: Bing**

| **#** | **Sites** | **Search** | **Results** | **Screened** | **Potentially relevant records** |
| --- | --- | --- | --- | --- | --- |
| 5a | (site:nhs.uk OR site:nhs.wales OR site:hscni.net) | (prehabilitation OR prehab) | 57,300 | 100 | 13 |
| 5b | (site:org.uk) |  | 12,600 | 100 | 3 |
| 5c | (site:ac.uk) |  | 17,300 | 100 | 0 |
| 5d | No restriction |  | 89,500 | 100 | 6 |
| 6a | (site:nhs.uk OR site:nhs.wales OR site:hscni.net) | (prehabilitation OR prehab) AND patient AND information | 1,160 | 100 | 2 |
| 6b | (site:org.uk) |  | 248 | 100 | 1 |
| 6c | (site:ac.uk) |  | 143 | 100 | 0 |
| 6d | No restriction |  | 3280 | 100 | 2 |
| 7a | (site:nhs.uk OR site:nhs.wales OR site:hscni.net) | (prehabilitation OR prehab) AND cancer | 1,270 | 100 | 5 |
| 7b | (site:org.uk) |  | 165 | 100 | 3 |
| 7c | (site:ac.uk) |  | 233 | 100 | 0 |
| 7d | No restriction |  | 3,630 | 100 | 1 |
| 8a | (site:nhs.uk OR site:nhs.wales OR site:hscni.net) | (prehabilitation OR prehab) AND patient AND information AND cancer | 1,220 | 100 | 2 |
| 8b | (site:org.uk) |  | 221 | 100 | 5 |
| 8c | (site:ac.uk) |  | 226 | 100 | 0 |
| 8d | No restriction |  | 2,870 | 100 | 1 |
