# Supplementary material for: Promoting or pressurising participation? A discourse analysis of online patient information resources about prehabilitation before cancer treatment
Source: PLoS One. 2025 Dec 23;20(12):e0339169. doi: 10.1371/journal.pone.0339169 (PMC12725656; doi:10.1371/journal.pone.0339169)
Supplement: S2 Appendix — (DOCX) [file pone.0339169.s002.docx]

| **SOURCE** | **Authorship** | **Attribution** | **Attribution 2** | **Conflict of Interest** | **Currency** | **Complementarity** | **Tone** | **Total** |
| --- | --- | --- | --- | --- | --- | --- | --- | --- |
| NHS Trust 1 | 0 | 0 | 0 | 2 | 0 | 1 | 0 | **7** |
| NHS Trust 2 | 0 | 0 | 0 | 2 | 0 | 1 | 0 | **7** |
| NHS Trust 3 | 0 | 0 | 0 | 2 | 0 | 1 | 0 | **7** |
| Cancer Alliance 1 | 0 | 0 | 0 | 2 | 0 | 1 | 0 | **7** |
| NHS Trust 4 | 0 | 0 | 0 | 2 | 0 | 1 | 0 | **7** |
| Charity 1 | 0 | 0 | 0 | 2 | 0 | 1 | 0 | **7** |
| NHS Trust 5 | 0 | 0 | 0 | 2 | 2 | 1 | 0 | **9** |
| NHS Trust 6 | 0 | 0 | 0 | 2 | 2 | 1 | 0 | **9** |
| Partner 1 | 0 | 1 | 0 | 2 | 0 | 1 | 0 | **10** |
| NHS Trust 7 | 0 | 1 | 0 | 2 | 0 | 1 | 0 | **10** |
| Charity 2 | 0 | 1 | 0 | 2 | 0 | 1 | 0 | **10** |
| NHS Trust 8 | 0 | 1 | 0 | 2 | 0 | 1 | 0 | **10** |
| NHS Trust 9 | 0 | 1 | 0 | 2 | 0 | 1 | 0 | **10** |
| NHS Trust 10 | 0 | 1 | 0 | 2 | 0 | 1 | 0 | **10** |
| NHS Trust 11 | 0 | 1 | 0 | 2 | 0 | 1 | 0 | **10** |
| NHS Trust 12 | 0 | 1 | 0 | 2 | 0 | 1 | 0 | **10** |
| NHS Trust 13 | 0 | 1 | 0 | 2 | 0 | 1 | 0 | **10** |
| NHS Trust 14 | 0 | 1 | 0 | 2 | 0 | 1 | 0 | **10** |
| Partner 2 | 0 | 1 | 0 | 2 | 0 | 1 | 0 | **10** |
| NHS Trust 15 | 0 | 1 | 0 | 2 | 0 | 1 | 0 | **10** |
| NHS Trust 16 | 0 | 1 | 0 | 2 | 0 | 1 | 0 | **10** |
| NHS Trust 17 | 0 | 1 | 0 | 2 | 0 | 1 | 0 | **10** |
| Partner 3 | 0 | 1 | 0 | 2 | 0 | 1 | 0 | **10** |
| Partner 4 | 0 | 1 | 0 | 2 | 0 | 1 | 0 | **10** |
| Partner 5 | 0 | 1 | 0 | 2 | 0 | 1 | 0 | **10** |
| NHS Trust 18 | 0 | 1 | 0 | 2 | 0 | 1 | 0 | **10** |
| Cancer Alliance 2 | 0 | 1 | 0 | 2 | 0 | 1 | 0 | **10** |
| NHS Trust 19 | 0 | 0 | 0 | 2 | 0 | 1 | 1 | **10** |
| NHS Trust 20 | 0 | 1 | 0 | 2 | 0 | 1 | 0 | **10** |
| NHS Trust 21 | 0 | 1 | 0 | 2 | 0 | 1 | 0 | **10** |
| NHS Trust 22 | 0 | 1 | 0 | 2 | 0 | 1 | 0 | **10** |
| NHS Trust 23 | 0 | 0 | 0 | 2 | 0 | 1 | 1 | **10** |
| NHS Trust 24 | 0 | 1 | 0 | 2 | 0 | 1 | 0 | **10** |
| Charity 3 | 0 | 0 | 0 | 2 | 0 | 1 | 1 | **10** |
| Cancer Alliance 3 | 0 | 1 | 0 | 2 | 0 | 1 | 0 | **10** |
| Cancer Alliance 4 | 0 | 1 | 0 | 2 | 0 | 1 | 0 | **10** |
| Charity 4 | 0 | 1 | 0 | 2 | 0 | 1 | 0 | **10** |
| Charity 5 | 0 | 1 | 0 | 2 | 2 | 1 | 0 | **12** |
| NHS Trust 25 | 0 | 1 | 0 | 2 | 2 | 1 | 0 | **12** |
| NHS Trust 26 | 0 | 1 | 0 | 2 | 2 | 1 | 0 | **12** |
| NHS Trust 27 | 0 | 1 | 0 | 2 | 2 | 1 | 0 | **12** |
| Charity 6 | 0 | 1 | 0 | 2 | 2 | 1 | 0 | **12** |
| Charity 7 | 0 | 1 | 0 | 2 | 2 | 1 | 0 | **12** |
| NHS Trust 28 | 0 | 1 | 0 | 2 | 2 | 1 | 0 | **12** |
| Cancer Alliance 5 | 0 | 1 | 0 | 2 | 2 | 1 | 0 | **12** |
| NHS Trust 29 | 0 | 1 | 0 | 2 | 2 | 1 | 0 | **12** |
| NHS Trust 30 | 0 | 1 | 0 | 2 | 2 | 1 | 0 | **12** |
| NHS Trust 31 | 0 | 1 | 0 | 2 | 2 | 1 | 0 | **12** |
| NHS Trust 32 | 0 | 1 | 0 | 2 | 0 | 1 | 1 | **13** |
| Professional 1 | 1 | 1 | 0 | 2 | 2 | 1 | 0 | **13** |
| NHS Trust 33 | 1 | 1 | 0 | 2 | 2 | 1 | 0 | **13** |
| NHS Trust 34 | 0 | 1 | 0 | 2 | 0 | 1 | 1 | **13** |
| NHS Trust 35 | 1 | 1 | 0 | 2 | 2 | 1 | 0 | **13** |
| NHS Trust 36 | 0 | 1 | 0 | 2 | 0 | 1 | 1 | **13** |
| NHS Trust 37 | 0 | 1 | 0 | 2 | 0 | 1 | 1 | **13** |
| NHS Trust 38 | 1 | 1 | 0 | 2 | 2 | 1 | 0 | **13** |
| NHS Trust 39 | 1 | 1 | 0 | 2 | 2 | 1 | 0 | **13** |
| NHS Trust 40 | 1 | 1 | 0 | 2 | 2 | 1 | 0 | **13** |
| NHS Trust 41 | 0 | 1 | 0 | 2 | 2 | 1 | 1 | **15** |
| NHS Trust 42 | 0 | 1 | 0 | 2 | 2 | 1 | 1 | **15** |
| Charity 8 | 2 | 1 | 0 | 2 | 2 | 1 | 1 | **17** |
| Charity 9 | 2 | 2 | 0 | 2 | 2 | 1 | 0 | **17** |
| NHS Trust 43 | 2 | 2 | 0 | 2 | 2 | 1 | 0 | **17** |
| NHS Trust 44 | 0 | 1 | 0 | 2 | 2 | 1 | 2 | **18** |
| Partner 6 | 0 | 3 | 2 | 2 | 0 | 1 | 0 | **18** |
| Charity 10 | 2 | 3 | 0 | 2 | 2 | 1 | 1 | **23** |
| Charity 11 | 0 | 3 | 2 | 2 | 2 | 1 | 2 | **26** |
| Information 1 | 2 | 3 | 2 | 2 | 2 | 1 | 2 | **28** |
