# Supplementary material for: Promoting or pressurising participation? A discourse analysis of online patient information resources about prehabilitation before cancer treatment
Source: PLoS One. 2025 Dec 23;20(12):e0339169. doi: 10.1371/journal.pone.0339169 (PMC12725656; doi:10.1371/journal.pone.0339169)
Supplement: Attachment — Submitted filename: Response to Reviewers.docx [file pone.0339169.s004.docx]

Dear Editor,

We thank the reviewers for their insightful comments on our manuscript entitled *‘Promoting or pressurising participation? A discourse analysis of online patient information resources about prehabilitation before cancer treatment’.* We have revised our manuscript in light of these comments. Please find a point-by-point explanation of our changes detailed in the table below.

We would also like to update our funding statement and acknowledgements as advised.

Our new funding statement reads:

*“SS is an Academic Clinical Fellow whose salary is part funded by the National Institute for Health and Care Research (NIHR). The salary for HS was funded by the NIHR Health Services and Delivery Research programme as part of the PARITY study (NIHR134282). The views expressed are those of the authors and not necessarily those of the NIHR or the Department of Health and Social Care. The funder did not play any role in the study design, data collection and analysis, decision to publish, or preparation of the manuscript. There was no additional external funding received for this study.”*

Our amended acknowledgements section reads:

*“The authors thank Lancaster University librarian John Barbrook for his advice on searching for grey literature. No other competing interests declared.”*

We hope the reviewers are satisfied with our changes and once again we thank them for their time in commenting on our manuscript.

Kind Regards,

Sophie Stanley

| **Reviewer Comment** | **Response** |
| --- | --- |
| Abstract: “Databases, search engines and websites (identified by experts)”…. Who is an expert? | Experts are members of the PARITY study team who conducted a survey of prehabilitation services in the UK. We have clarified this in the abstract by amending the text to read *“identified by prehabilitation researchers”*. |
| This appears to be a systematic review, but the authors have not provided any evidence of prospective registration of the protocol. Hence, it is not possible to decide if the students followed the predetermined protocol. This makes it PRISMA non-compliant. Please clarify. | This is not a systematic review. We have applied systematic review search methods to online grey literature as outlined by Godin et al. This enabled us to systematically search for online patient information materials. The aim of this work was not to synthesise these resources (as would be the case in a systematic review) but to use these documents as primary data for qualitative discourse analysis. |
| The Introduction runs into 4 pages and should be made more succinct. On the whole, the paper is very long and could be abridged. | The introduction has been reduced to just over 2 pages. Some sections of the method and discussion have been reduced also. |
| Why did the authors limit their search to UK data? This limits generalisability. | Discourse analysis is the study of language in its social and cultural context. On that basis, we chose to conduct this study within a common language (e.g. UK English) and a ‘national’ service (e.g. the National Health Service). This provided a relatively consistent social and cultural context in which to study the language used about prehabilitation. We have explained this rationale within the manuscript by including the following text:  *“Discourse analysis is the study of language in its social and cultural context. We chose to conduct this study within a common language (e.g. UK English) and a ‘national’ service (e.g. the National Health Service) to ensure socio-cultural and linguistic consistency.”* |
| There is no data-sharing statement. | We will include the following data sharing statement:  *“The data used in this study are already publicly available. We sought to protect the identities of the organisations who produced this data. We have chosen not to share openly the individual resources or websites analysed as this would compromise that aim.* *Further information about the data used and conditions for access are available from Lancaster University's Institutional Repository at* [DOI to be provided]*”* |
| All included studies/resources have not been referred to in the bibliography | We have not referenced the individual patient information resources that formed the primary data for our discourse analysis. As mentioned in our ethics statement, we sought to protect the identities of the institutions producing these resources, focusing on their content instead. |
